# Protecting aquatic biodiversity in Europe: How much do EU environmental policies support ecosystem-based management?

**DOI:** 10.1007/s13280-017-0928-4

**Published:** 2017-06-13

**Authors:** Josselin Rouillard, Manuel Lago, Katrina Abhold, Lina Röschel, Terri Kafyeke, Verena Mattheiß, Helen Klimmek

**Affiliations:** 1 Ecologic Institute, Pfalzburgerstr. 43/44, 10717 Berlin, Germany; 2 ACTeon, 5 Place Sainte-Catherine, 68000 Colmar, France; 3 IUCN, 64 Boulevard Louis Schmidt, 1040 Brussels, Belgium

**Keywords:** Aquatic ecosystems, Ecosystem-based management, EU policy, Evaluation, Nature protection

## Abstract

The sustainable management of aquatic ecosystems requires better coordination between policies span-ning freshwater, coastal and marine environments. Ecosystem-based management (EBM) has been promoted as a holistic and integrative approach for the safekeeping and protection of aquatic biodiversity. The paper assesses the degree to which key European environmental policies for the aquatic environment, namely the Birds and Habitats Directives, Water Framework Directive and Marine Strategy Framework Directive, individually support EBM and can work synergistically to implement EBM. This assessment is based on a review of legal texts, EU guidance and implementation documents. The paper concludes that EBM can be made operational by implementing these key environmental directives. Opportunities for improving the integration of EU environmental policies are highlighted.

**Electronic supplementary material:**

The online version of this article (doi:10.1007/s13280-017-0928-4) contains supplementary material, which is available to authorized users.

## Introduction

Biodiversity is declining worldwide, and at a much faster rate in aquatic than in most terrestrial systems (Vaughn 2010). Political action at regional and global levels has sought to curb such trends. In Europe, the implementation of the Birds and Habitat Directives (“the Nature Directives”), the Water Framework Directive (WFD) and the Marine Strategy Framework Directive (MSFD) aim to protect aquatic biodiversity and environments. More recently, the EU 2020 Biodiversity Strategy aims to implement the Strategic Plan for Biodiversity 2011–2020 and the Aichi Targets (CBD-UNEP 2010, 2013).

Despite progress, EU directives have been unable to halt and reverse the trend of declining biodiversity of aquatic ecosystems in Europe (EEA 2015). As a result, the EU is seeking new approaches to achieve the EU Biodiversity Targets, including by improving the coherence between the Nature Directives, the WFD and MSFD (CIS 2013a, b, 2015) so as to provide better biodiversity protection across the freshwater, coastal and marine continuum. In parallel, the concept of ecosystem-based management (EBM) has been promoted in academic circles to support more effective implementation of environmental and water policies (Apitz et al. 2006; Vlachopoulou et al. 2014). In particular, EBM aims to create management systems that better protect the dynamics and requirements of healthy ecosystems.

To date, research studies have evaluated the implementation of EBM against singular policies or within isolated aquatic ecosystems. The majority of studies have focused on marine systems and the analysis of EBM in the MSFD (Holt et al. 2011; Raakjaer et al. 2014; Berg et al. 2015; Soma et al. 2015; Nilsson and Bohman 2015). Research in freshwater systems has focused on the ecosystem “approach” in the WFD (Kallis and Butler 2001; Borja et al. 2010; Vlachopoulou et al. 2014), while the link between EBM and the Nature Directives has mostly been examined in the context of conservation activities in marine systems (e.g. Clarke et al. 2003; Holt et al. 2011). As such, no papers have evaluated how EBM can be implemented horizontally across the Nature Directives, the WFD and MSFD, and how it can inform and support policy integration between these policies.

This paper evaluates the possible future use of EBM as an integrative policy concept for the protection of aquatic biodiversity in Europe. It examines the degree to which the Nature Directives, WFD and MSFD align with EBM principles and how they can work synergistically to implement EBM and protect aquatic biodiversity across realms. This exercise enables to highlight the synergies, barriers and opportunities between water-, marine- and nature-relevant policies for more effective implementation of environmental protection policies across aquatic ecosystems in Europe. Insights gained in this paper can thus inform not only current academic discussions on EBM implementation but also policy developments into the streamlining and coordinated implementation of freshwater, coastal and marine policies.

The paper first outlines the methodological approach, presenting policy-relevant EBM principles and the approach taken to assess the four policies. The paper then presents the detailed assessment made on the Nature Directives, WFD and MSFD, followed by a discussion on synergies and key areas of mismatch and conflict. We conclude on the scope for the EBM to work as an integrative policy tool for the protection of aquatic ecosystems in Europe.

## Materials and methods

### The EU policy framework for the protection of aquatic biodiversity

A large number of European policies can directly or indirectly impact aquatic biodiversity. Such policies may include “emission control” policies, such as the Nitrates Directive (91/676/EEC), “sectoral” policies, such as the Common Fisheries Policy (CFP) (380/2013), and general “growth” and infrastructure development policies, such as cohesion and structural funds. However, four directives have proved to be most significant as they establish a holistic approach to the protection of aquatic biodiversity across freshwater, coastal and marine realms.

The Birds and Habitats Directives are often referred to jointly as their design and implementation are closely related. The Birds Directive (BD) (79/147/EC) aims to protect all wild bird species naturally occurring within the EU, while the Habitats Directive (HD) (92/43/EEC) aims to conserve natural habitats and wild fauna and flora in the European territory of the Member States to which the treaty applies. The WFD (2000/60/EC) aims to promote long-term sustainable water management based on a high level of protection of the aquatic environment. The MSFD’s (2008/56/EC) objective is to protect and preserve the marine environment, prevent its deterioration and restore the environment in areas where it has been adversely affected. Together, these four environmental Directives provide the legislative foundation to protect aquatic biodiversity across the freshwater–marine continuum and thus the most relevant pieces of enabling legislation for EBM.

Though sectoral policies such as the CFP and Common Agricultural Policy (CAP), as well as the other emissions control policies and growth and industrial development policies mentioned above are relevant to the successful implementation EBM, this paper’s aim is to assess the extent to which principles of EBM are represented in key EU environmental policies that underpin Member State obligations to protect aquatic biodiversity. This is an important first step to analyse the political framework enabling EBM of aquatic ecosystems.

### Assessing EBM in European environmental policies

EBM is a complex concept, incorporating a wide range of principles. Though the concept of EBM has taken root in the political sphere, there is currently no single, agreed-upon overarching definition of EBM. However, it can generally be understood as any management or policy option intended to restore, enhance and/or protect the resilience of an ecosystem so as to sustain or improve the flow of ecosystem services and conserve biodiversity. This includes any course of action purposely intended to improve the ability of an ecosystem to remain within critical thresholds, to respond to change and/or to transform to find a new equilibrium or development path (Gómez et al. 2016).

Borgström et al. (2015) developed a generic EBM analytical framework that aimed to link ecosystem aspects with specific phases of the management cycle. The authors’ ultimate goal was to create an analytical assessment tool that simultaneously assessed ecological goals and ambitions, as well as social processes, management strategies and actions. Our research faced the specific challenge of examining EBM at the level of policies. Reviewing existing work on EBM in aquatic ecosystems, such as work done on defining EBM for the governance of aquatic ecosystems by Gómez et al. (2016), marine systems by Long et al. (2015), meta-ecosystems by Loreau et al. (2003) and the Ecosystem Approach concept as discussed by the Convention on Biological Diversity (CBD-UNEP 2011), we developed a consolidated definition of EBM consisting of six policy-relevant principles (Table 1).

**Table 1:** Policy-relevant principles of EBM

EBM principle	Description
1. EBM considers ecological integrity, biodiversity, resilience and ecosystem services	Focuses on multiple ecosystem services and aims to maximise their join value Considers the dynamic relationships within ecosystems
2. EBM is carried out at appropriate spatial scales	Considers ecosystem rather than jurisdictional boundaries to reach decisions and take actions at the appropriate level Considers complex and adaptive processes May require transboundary cooperation
3. EBM develops and uses multi-disciplinary knowledge	Requires a multi-disciplinary approach Relies on a detailed understanding of the social-ecological system, drawing on scientific as well as local and traditional knowledge
4. EBM builds on social–ecological interactions, stakeholder participation and transparency	Acknowledges social–ecological interactions and seeks to balance ecological and social concerns Considers synergies and trade-offs between benefits and beneficiaries Gives preference to transparent and inclusive decision making Seeks to build consensus on a shared vision for the future
5. EBM supports policy coordination	Facilitates cooperation and collective action across different stakeholder and policy domains to share the array of ecosystem services obtained Creates new opportunities to pursue different policy objectives simultaneously
6. EBM incorporates adaptive management	Aims to increase adaptive capacity by restoring critical ecosystems and strengthening social capacities to respond to a range of possible future scenarios Weighs short-term management options against long-term benefits of alternative interventions Monitors impact and regularly revisits management tools

Using this consolidated definition, we examined the degree to which key EU environmental policies relevant to the protection of aquatic biodiversity, namely the Nature Directives, the WFD and MSFD can support EBM implementation. The paper focuses on the legal and policy framework established at EU level to protect aquatic biodiversity through legislative instruments (e.g. EU Directives and related Regulations and Decisions) as well as less coercive instruments (e.g. related Communications and Guidance documents). The analysis compared the legislative text of the four Directives against the six EBM principles, undertaking a cross-analysis to assess the extent to which each Directive reflected elements of these principles as well as how well-represented these principles were throughout all four Directives. The strength and weaknesses of each Directive were assessed against each principle, as presented in the Supplementary Material (hereafter referred to as SM S1).The research thus concentrates on assessing the degree of consistency between EBM principles and requirements and incentives set by EU policies in different areas, including objectives and targets, planning steps and scales, and management measures (Howlett 2009). In addition to the legislative and policy texts of the Directives, a number of supporting sources were used to ensure adequate interpretation of the legislation, including relevant policy and implementation reports produced by EU institutions (e.g. European Commission, European Environmental Agency) (see list in the SM S1).

The results section presents an overview of the outcomes of this assessment. Additional information is provided in the SM S1. In the discussion section, we use these results to compare the different environmental policies and their ability to work synergistically or antagonistically for the implementation of EBM.

## Results

### The Nature Directives

The aim of the HD is to maintain and restore all habitat types and species of community interest to a Favourable Conservation Status (FCS). FCS describes a situation where a habitat type or species is prospering in both quality and extent and population, and has good prospects to do so in the future. The BD focuses on conserving all naturally occurring birds in the wild. The BD calls for measures to protect birds but also to preserve, maintain (prevent deterioration) or re-establish a sufficient diversity and area of habitats for certain bird species. A pre-defined list of habitats and species are set out in the directives. These measures focus on biodiversity and have the potential to have a positive impact on the whole ecosystem (EBM Principle 1). However, neither Directive explicitly mentions ecosystem services nor take them into account implicitly. Recent work has nevertheless mapped habitats and species classified under the Nature Directives with ecosystem types of MAES (Mapping and Assessment of Ecosystems and their Services) (EEA 2015).

The Directives also acknowledge the multi-level approach to biodiversity conservation by enabling proportionate and appropriate implementation in each State and at site level (EBM Principle 2). While protecting species across their entire natural range, both Directives support the establishment of a network of protected areas to protect the most vulnerable species and habitat types, commonly called together as Natura 2000. Internationally, the Directives acknowledge that threats to habitats and species are often of a transboundary nature, and explicitly call for cooperation between Member States. At local level, they encourage the use of management, contractual agreement between the competent authorities and individual landowners (EC 2000).

The development of a protection regime for habitats and species, and designation of Natura 2000 sites, is done on scientific grounds and must consider elements of biology, ecosystem functions and structure (EBM principle 3). Under the HD, any plan or project likely to have a significant effect on a Natura 2000, either individually or in combination with other plans or projects, shall undergo an appropriate assessment to determine its implications for the site. While effects of biodiversity loss, habitat fragmentation and ecological dynamics are considered, there is no specific requirement to identify and consider key thresholds in social–ecological dynamics in order to maintain ‘resilience’. Both Directives include nevertheless consideration of social and economic issues, whereby States must provide information on threats and pressures (Art. 12 BD, Art. 17 HD). Measures must take into account economic, social and cultural requirements and regional and local characteristics of the area concerned (Art. 2 HD and BD).

Regarding public participation (EBM principle 4), Directive does not require the active involvement of stakeholders and inclusion of community knowledge. Official EU guidance nevertheless encourages States to involve the public, e.g. on issues related to the establishment of the conservation measures (EC 2012a, b, c). Member States are also asked to reflect on positive changes in public acceptance towards biodiversity protection, and cooperation between authorities, nature conservationists and other interest groups and initiatives. Furthermore, under the BD, States may derogate in the interest of public health or safety, air safety, for the protection of flora and fauna and to prevent damage to crops, livestock, fisheries and water (Art. 9).

In terms of policy coordination (EBM principle 5) between the BD and the HD, the protection regime between protected areas was harmonised through Art. 7 of the HD (Milieu et al. 2015). A change from a 3- to 6-year reporting cycle for the BD means that the BD and HD are now reasonably synchronised. Both directives are characterised by a similar dual structure of measures with similar steps. Being anterior to the WFD and MSFD, there is no specific requirement in the Nature Directives to coordinate with the water and marine legislation. The HD requires adoption of prioritised action frameworks (Art. 8) to define the funding needs and priorities for Natura 2000 at a national or regional level, which facilitate integration into other EU instruments. Thus, theoretically, funding appears to be available and to a degree coordinated between different policy instruments.

Lastly, the Nature Directives require Member States to report progress on the state of conservation every six years. While this encourages some cycles of planning and revisions, it is not clearly spelled out in both directives. States also have a certain margin of manoeuvre or flexibility in implementing provisions. Under the HD, Member States can propose adaptations to the list of Special Areas of Conservation (SACs) in light of results of surveillance of conservation status of habitats and species (Art. 6). The concrete targets to be achieved can vary and can also evolve with for example better scientific knowledge. Finally, the HD stresses for example the need to go beyond simple management measures to ensure conservation towards preventive and anticipatory approaches to avoid deterioration, which can build adaptive capacity and resilience (EBM principle 6). More information can be found for the Nature Directives in ESM-S1.

### The Water Framework Directive

The key objective of the WFD is to achieve good status or potential for all water bodies by 2015 and avoid deterioration (Art. 4). Ecological status is an expression of the quality of the structure and functioning of aquatic ecosystems associated with surface waters. It is defined as the deviation of specified biological elements from undisturbed reference conditions, supported by hydromorphological and physicochemical quality elements. Furthermore, through status classes, the WFD acknowledges the need to maintain ecosystems within certain ranges to maintain ecological integrity (EBM principle 1). The WFD does not explicitly integrate the notion of resilience and ecosystem services, although recent policy developments emphasise the need to realise multiple benefits (EC 2012b).

Pertaining to the spatial components (EBM principle 2), the WFD sets the primary management units at the level of hydrological water bodies and the administrative unit at the level of river basin districts (RBDs), including transboundary ones. All rivers, lakes, estuaries, groundwater and coastal waters out to one nautical mile (12 nautical miles for chemical status) fall within the scope of the WFD. The WFD promotes integrated water and land management, and therefore has expanded the traditional scale of water management from a sole focus on aquatic systems to surrounding land. The scales promoted by the WFD may not always be appropriate to tackle the threats to the relevant aquatic ecosystem, for example when needing to tackle nitrogen deposition from air pollution or when considering migratory fish with the open seas.

The characterisation of the RBD (Art. 5) includes an analysis of pressures and impacts from human activities, the economic analysis, the delineation of water bodies and the establishment of the typology and reference conditions for surface water bodies. The selection of measures has to take cost-effectiveness into account, and ensure compliance at minimum costs for both public and private entities. Overall, the WFD requires the mobilisation of knowledge from different scientific disciplines (e.g. ecology, chemistry, economy) (EBM principle 3). However, the WFD does not ask for a detailed understanding of ecosystem functions and structures, nor does it specify how stakeholder opinions and knowledge should be taken into account.

While the objective of good ecological status requires adequate attention to ecological needs, socio-economic concerns are considered in several ways (EBM principle 4). The active involvement of all interested parties in implementation is for example required, in particular the production, review and updating of river basin management plans (RBMPs) (Art. 14). Authorities must publish openly the timetables, assessment reports and draft RBMPs. The use of exemptions to reaching the environmental objectives is also possible if certain conditions are met. Exemptions include extension of deadlines (Art. 4.4), less stringent objectives (Art. 4.5), temporary deterioration (Art. 4.6) and new modifications (Art. 4.7).

Within the WFD, integrated water management and policy coordination are explicit aims (EBM principle 5). The WFD specifically harmonises objectives and approaches across water-related policies by requiring the inclusion of relevant measures from other water directives in the WFD Programme of Measures (PoM). Because the WFD is anterior to the MSFD, it does not create specific linkages with the MSFD, although it generally requires that implementation should contribute to the protection of marine waters (Art. 1). The WFD does, however, provide more specific linkages with the Nature Directives, such as compliance with standards and objectives (Art. 4.9) (EC 2011a, b, c). In any cases, exemptions under the WFD must be coherent with the measures taken under the Nature Directives which take precedence (Art. 4.9). Recent initiatives at EU level such as Natural Water Retention Measures promote integrated measures across the WFD, Flood Directive, Nature Directives and others (EC 2011b).

The WFD integrates several aspects of adaptive management (EBM principle 6). The WFD is organised around a six-year planning cycle, which include a thorough evaluation of the success of past implementation. Up to three planning cycles (by 2027 at the latest) are allowed to reach the environmental objectives. Protection and restoration measures on ecosystems are at the core of the WFD, while preventive measures are promoted, for example efficient water use and the prevention of accidental pollution (Art. 11.3). The WFD mentions the precautionary principle and does not allow deterioration in the status of water bodies (unless exemptions apply) (Art. 1). These measures can increase resilience and robustness and form part of a strategy to deal with uncertain future events. The WFD does however not integrate climate change in its legal text, although it can be integrated into the planning process (EC 2009). More information can be found for the Nature Directives in ESM-S2.

### The Marine Strategy Framework Directive

The MSFD includes explicitly and implicitly the concepts of “ecological integrity”, “biodiversity”, “resilience” and “ecosystem services” (EBM principle 1). The overall objective of the MSFD is to establish a framework to achieve or maintain Good Environmental Status (GES) in the marine environment by the year 2020 at the latest. GES is determined on the basis of 11 qualitative descriptors, including biodiversity, ecological integrity, safe biological limits and others (set out in Annex 1 of the Directive). GES is associated with a situation whereby the structure, functions and processes of marine ecosystems allow those ecosystems to function fully and maintain resilience. Importantly, Member States must apply the ecosystem approach to keep levels of human activities compatible with the achievement of GES (Art. 1.3).

Spatially (EBM principle 2), the MSFD covers marine waters (the waters, seabed and subsoil) of Member States’ jurisdictional reach and coastal areas (Art. 3.1). However, environmental status may include factors that may affect the area both from within and outside the area concerned (Art. 3.4). The MSFD thus establishes marine regions that go beyond Member States’ territorial boundaries. Member States should not only consider other nations’ territories as extension of their own ecosystems, but should evaluate how they themselves affect marine areas that lie beyond their borders (Art. 13.8). There is thus much emphasis in the MSFD on transboundary cooperation from Member States, in particular regarding monitoring and implementation of measures.

The MSFD calls for Member States to undertake a number of multi-disciplinary assessments (EBM principle 3) as part of the planning process, including environmental status and socio-economic features of their marine environments (Art. 8.1). Planning steps include an analysis of pressures and impacts of the marine environment. Member States are further required to consider the social and economic impacts of measures to reach environmental objectives, through for example cost–benefit analysis and cost-effectiveness analysis (Art. 13.3) (EC 2013).

The MSFD provides a comprehensive framework for considering social–ecological interactions (EBM principle 4). Member States should make scientific information on the intended affects of their PoMs available to the general public (Art. 13.6) and must offer opportunities to interested parties to participate (Art. 19.1), in particular people most affected by changes in ecosystem services (EC 2011c). Furthermore, Member States are allowed to adopt derogations in the form of “exceptions” to reaching the environmental targets due to modifications or alterations brought about by actions taken for reasons of overriding public interest which outweigh the negative impact on the environment (Art. 14.1). In addition, Member States are not required to take action if the costs to achieve GES are deemed ‘disproportionate’ (Art. 14.4) (see EC 2015a, b for examples).

With regards to policy coordination (EBM principle 5), the MSFD legal text explicitly makes reference to multiple policies and their coordination (Art. 13.2). Types of measures suggested by the MSFD and supported by the CIS include management coordination measures (EC 2015a, b). Annex IV of the MSFD highlights that environmental targets must be compatible with existing commitments, including those under the Nature Directives and WFD. Thus, implementation of MSFD cannot impair the implementation of the Nature Directives, and the application of “exceptions” under the MSFD cannot take precedence over Nature Directives obligations (EC 2012c). In other words, FCS is a regulatory minimum under the MSFD.

The MSFD explicitly incorporates adaptive management (EBM principle 6) in Art. 3.5. Member States must regularly update their marine environment assessments, their targets for GES, monitoring programmes and PoMs every six years (Art. 17). The directive promotes a precautionary approach so that the capacity of marine ecosystems to respond to human-induced changes is not compromised (i.e. resilience) (Art. 1.3). Attainment or maintenance of good environmental status is seen as maintaining ecosystem resilience (Art. 3.5). The MSFD does not set out an explicit approach to manage uncertainties, and Member States are not required to adopt mitigation measures to respond to expected long-term changes, such as climate change. Follow-up guidance suggests nevertheless that sources of uncertainty should be explicitly identified, especially during the economic and social analysis (EC 2011c). More information can be found for the Nature Directives in ESM-S3.

## Discussion

Building on the overview of how individually the Nature Directives, WFD and MSFD support EBM, the discussion aims to answer the following question: how much can they work together to support each EBM principle? This analysis also provides insights into key synergies, mismatches and conflicts between the four directives. Table 2 provides an overview of strengths and weaknesses.

**Table 2:** Strength and weaknesses in the coordination of the Nature Directives, WFD and MSFD in relation to key EBM principles

EBM principle	Strengths	Weaknesses/challenges
1: EBM considers ecological integrity, biodiversity, resilience and ecosystem services	Reviewed policies support the key concepts of EBM implicitly, with undisputed linkages in their objectives with biodiversity conservation	No clear policy framework for taking into account ecosystem services and managing trade-offs, which reduces the potential effectiveness of the policy instruments towards biodiversity protection. The WG MAES framework could be applied to streamline approaches among the Directives
2: EBM is carried out at appropriate spatial scales	Management is encouraged at relevant ecological scales, while multiple levels in social systems (and the need to coordination) are acknowledged	No clear framework or guidance on how to work across scales; no clear acknowledgment of cross water realms linkages (except in MSFD); objectives set a specific scales (e.g. water body level in WFD) may not take into account of ecological dynamics
3: EBM develops and uses multi-disciplinary knowledge	Reviewed directives encourage inter-disciplinary approaches and consideration of societal values and interest in decision making	No explicit requirement to integrate local knowledge (e.g. to improve contextual understanding of management units) Differences in objectives, scope and approaches result in different monitoring needs. Synergies in monitoring programmes can be exploited. The main objective should be to integrate monitoring as far as possible
4: EBM builds on social–ecological interactions, stakeholder participation and transparency	Participation is an element of all reviewed directives and mechanisms are crafted to enable a balance between ecological and social concerns	Unclear distribution of powers and role of local communities in decision making (e.g. who decides?) Multiple types of criteria for derogations among directives which increase potential for different interpretation and conflicts
5: EBM supports policy coordination	Policy coordination is strongly encouraged Scope for revisions of the legal acts to foster further policy integration in line with Biodiversity Strategy objectives Scope for funding instruments to support integration of Programme of Measures	Few specific mechanisms that help strong coordination are proposed, especially outside protected areas
6: EBM incorporates adaptive management	Policies support evaluation of management measures, with clear (although separate) planning cycles for HD&BD, WFD and MSFD	No strong framework for dealing with uncertainties (and climate change), no legislative guidance with regards to timescale envisaged, limited length of regulatory requirements (e.g. WFD revisions in 2020s) and no clear methodological proposition (e.g. use of scenarios)

With regards to the EBM principle 1, the focus of the nature, water and marine environmental policies is on species diversity, protection of key species and habitats, and reaching environmental state indicators, which are closely linked to biodiversity conservation and maintenance of ecological integrity. The MSFD also explicitly includes the concept of ecosystem services provision, while safeguarding the overall (not just some) provision of ecosystem services which is not a stated objective in the Nature Directives and WFD. Fundamentally, the implementation of nature and water Directives in isolation is mainly focused on certain ecosystem services (e.g. maintain nursery populations and habitats under the HD and BD, drinking water provision under the WFD). Within EBM it would be important to lay synergies and conflicts in ecosystem services provision, and to let society prioritise between them. Fundamentally, there are always trade-offs, and choices have to be made about the species or habitats which shall be protected in priority.

Furthermore, there are cases where Nature Directives and WFD do not target overall biodiversity protection. For example, because the WFD looks at the presence or absence of certain species for the assessment of good ecological status, many of the WFD restoration actions can be targeted towards increasing the numbers of these species. However, the representative species that are selected as indicators may not be the ones that better reflect the structure and functioning of the ecosystem. Thus, the ability of the ecosystem to support biodiversity may be affected by the WFD actions. One example is how fish stocking (to meet WFD objectives) has been found, among other drivers and pressures, to have a negative impact in freshwater pearl mussel populations in the river Rede in the UK (Gosselin 2015).

With regard to EBM principle 2, the results highlighted that the Nature Directives protect natural terrestrial, freshwater and marine habitats (HD) and wild birds (BD), while the WFD targets freshwater and coastal waters, and the MSFD coastal and marine waters as well as the seabed and subsoil on which Member States have jurisdiction under international law. The Nature Directives overlap with both the WFD and MSFD, which calls for a number of harmonisation and coordination regarding objectives and targets, measures and exemptions (see below). There is a degree of equivalence between the WFD status categories and HD status classes (see EC 2015b), but there is no direct correspondence between WFD water body types and habitat types of the HD. Furthermore, the WFD and MSFD overlap in the one nautical mile from the shoreline. This calls for a need of harmonisation for those objectives that target similar pressures (e.g. eutrophication).

The nature, marine and water Directives all support the use of multi-disciplinary knowledge (EBM principle 3) to inform several aspects of their planning process; such as the understanding of threats, pressures and impacts to the environment. The four Directives do not require in-depth assessments of ecological functions and structures, but rather focus on drivers, pressures and state indicators which are linked to conditions that are deemed favourable for biodiversity. A vast amount of knowledge has been successfully mobilised in recent implementation cycles. However, much of this effort is focused on checking compliance towards objectives at the EU level rather than empowering management at the local level, as it can be seen by the lack of support to community knowledge. This highlights that the overall definition of knowledge that is used in these directives would have to be re-interpreted in order to better integrate different sources of knowledge and fit better with EBM principles.

The nature, water and marine directives acknowledge social–ecological interactions and the need to seek a balance between ecological and social concerns (EBM principle 4). All directives consider the costs and benefits of alternative courses of action to seek this balance. “Derogations” to the environmental objectives set out in the legal text are possible in all directives, in particular in cases of “overriding public interest”. In addition, the directives support transparent decision making to different degrees, via in particular the diffusion of information to the public and some form of consultation. However, the role of stakeholders and local actors is unclear in all four directives. There is no requirement to take into account the views expressed during consultation, and there is no requirement to create supporting institutional arrangements to tackle conflicting interests and advance collective action at local level.

With regards to policy coordination (EBM principle 5), the results suggest that there is potential for further integration. Currently, coordination between these policy areas is an implicit aim in WFD and MSFD legal texts. The MSFD in particular depends on the WFD for reducing pressures from freshwater and inland sources. However, mechanisms to enable integration with sectoral policies are not very strong and in most instances, they remain unclear. The WFD and MSFD both fully incorporate Nature Directives targets and measures, but this coordination is only a requirement when dealing with protected areas. Only the MSFD contains as a key objective that “biodiversity is maintained by 2020″ in close integration with the Biodiversity Strategy and it is the first EU legislation that aims at the protection of the full range of marine biodiversity as an integrative objective. The likely future revision of the WFD legal text offers a window of opportunity to ensure the inclusion of further provisions to streamline the WFD with the Nature and Marine Directives, under the umbrella of the Biodiversity Strategy objectives. In addition, the review and possible revision of the MSFD GES Decision 2010/477/EU could be used to integrate the approaches established under the WFD and the Nature Directives (CIS 2013a, b).

Lastly, adaptive management (EBM principle 6) through learning and adjustments are encouraged through monitoring and evaluation during planning cycles. All four Directives support preventing the loss of ecosystem resilience by reducing pressure and avoiding the deterioration of protected features (e.g. habitats, birds, water bodies, marine areas). While the directives mostly differ on their deadlines, they all have planning cycles of six years. They could thus be synchronised similar to that of the HD and BD in 2013. Overall, the four directives lack a long-term view (~50 to 100 years) and do not offer an explicit framework for dealing with uncertainties and future change. Uncertainty is dealt implicitly in a variety of ways, mostly by allowing flexibility in implementation or by applying the precautionary principle. Member States are not required to outline future scenarios and develop measures to respond to these scenarios, nor anticipate coordinated responses to risk events.

## Conclusion

Overall, there is a lot of EU policy support for the implementation of EBM and potential to increase synergies between policies with this purpose. The EU policy framework in the form of the Nature Directives, WFD and MSFD supports several key dimensions of EBM (e.g. ecological integrity, acknowledgement of multiple scales, multi-disciplinary knowledge, stakeholder participation, transparency, policy coordination, adaptive management). These commonalities represent opportunities for streamlining and coordinating between directives. Future research could investigate if the opportunities highlighted above are effectively exploited by implementing authorities and how. The policy review presented in this paper also highlights gaps in the four directives regarding several important dimensions of EBM, in particular with regards to: the implementation of the ecosystem services approach, the integration of planning processes and monitoring programmes, the integration of local knowledge in the decision-making process, coherent approaches to exemptions and derogations and the consideration of uncertainties in management and governance. Future work by EU policy-makers could focus on how to complement the current policy framework through more specific guidance or legislation on these dimensions. Further research could also investigate if implementing authorities have developed strategies to fill in or overcome these gaps. Thanks to a clear set of management principles, EBM is a useful concept to assess the implementation logic of European environmental policies and how they can work to protect aquatic biodiversity. Nevertheless, we hypothesise that, while EU environmental policies provide a sound legislative basis for implementing EBM, as demonstrated in this research, further streamlining and coordination across the wider spectrum of European policies would be needed to enable EBM in practice. Future research could thus expand the scope of the analysis presented in this paper and examine if the broader European policy framework, including economic and sectoral policies, supports EBM or not.

## Electronic supplementary material

Below is the link to the electronic supplementary material.

Supplementary material 1 (PDF 109 kb)
