# Supplementary material for: Protecting aquatic biodiversity in Europe: How much do EU environmental policies support ecosystem-based management?
Source: Ambio. 2017 Jun 13;47(1):15–24. doi: 10.1007/s13280-017-0928-4 (PMC5709260; doi:10.1007/s13280-017-0928-4)
Supplement: Supplementary file 1 — Supplementary material 1 (PDF 109 kb) [file 13280_2017_928_MOESM1_ESM.pdf]

Ambio

Electronic Supplementary Material (ESM)

This supplementary material has not been peer-reviewed.

Title: **Protecting aquatic biodiversity in Europe: how much do EU environmental policies support ecosystem-based management?**

Authors: Josselin Rouillard*, Manuel Lago, Katrina Abhold, Lina Röschel, Terri Kafyeke, Verena Mattheiß, Helen Klimmek

* Corresponding author, Email: josselin.rouillard@ecologic.eu

Appendix ESM S1: EBM principles reflected in the Birds and Habitats Directives

1. EBM considers ecological integrity, biodiversity, resilience and ecosystem services

Strengths

The overall objective of the Habitats Directive (HD) is to conserve natural habitats and wild fauna and flora in the European territory of the Member States. The HD also aims to maintain and restore to a Favourable Conservation Status (FCS) all habitat types and species of community interest. FCS describes a situation where a habitat type or species is prospering in both quality and extent and population - and has good prospects to do so in the future. The HD thus recognises that features of the landscape should be managed to support the conservation of relevant species. The Birds Directive (BD) focuses on conserving all naturally occurring birds in the wild state in the European territory of the EU Member States. The BD calls for measures to protect birds but also to preserve, maintain (prevent deterioration) or re-establish a sufficient diversity and area of habitats for certain bird species. These measures have the potential to have a positive impact not only on bird species but also on the wider ecosystem.

Recent work by the EEA has nevertheless mapped habitats and species classified under the Nature Directives against the working group Mapping and Assessment of Ecosystems and their Services (MAES) ecosystem types (EEA 2015). Some assessment also used the MAES and Common International Classification of Ecosystem Services (CICES) typology of Ecosystems and ecosystem services types to present progress in biodiversity protection in Europe through the Nature Directives. Other initiatives led by the EC, such as the application of Green Infrastructures, aims to provide a framework for the consideration of multiple benefits within nature protection, which aligns well with the ecosystem services concept.

Weaknesses

The Nature Directives do not consider explicitly ‘ecosystem services’.

While the Nature Directives do align with the idea that ecosystems should be protected so as to preserve an array of ecosystem services, their focus on protecting specific habitats and species does not necessarily align with the idea of maximising the joint value of all ecosystem services.

2. EBM is carried out at appropriate spatial scales

Strengths

The HD focus on terrestrial and aquatic habitats distinguished by geographic, abiotic and biotic features while the BD focuses on bird species. To support the conservation of habitats and species across their whole natural range, the Nature Directives require the establishment of a network of protected areas, commonly called Natura 2000. For the HD, Member States must propose a list of sites hosting habitats and species listed in the Annexes, which provides the basis for selection of Sites of Community Interest (SCI) and Special Areas of Conservation (SACs). For animal species ranging over wide areas the sites correspond to “the places within the natural range of such species which present the physical or biological factors essential to their life and reproduction. For aquatic species which range over wide areas, such sites will be proposed only where there is a clearly identifiable area representing the physical and biological factors essential to their life and reproduction” (Art. 4). Under the BD, Member States are free to designate the most suitable territories as SPAs for the conservation of species in the geographical sea and land area where this Directive applies. More generally, the BD establishes a general system of species protection in particular against hunting, trading and deliberate disturbance of bird species.

The Nature Directives also acknowledge the multi-level approach to biodiversity conservation by enabling proportionate and appropriate implementation in each Member State and at site level. The HD allows for flexibility in the type of conservation measures which have to be established for SACs, including "appropriate statutory, administrative or contractual measures' and 'if need be management plan'" (Art. 6). Under the BD, measures relate to protection of specific species (e.g., probation of hunting, capture), but also to protection of habitats, while the designation of SPAs contribute to Natura 2000, indicating conservation action at multiple scales.

Measures in Natura 2000 will involve different spatial scales. At local level, management agreement (Annex II A) will involve contractual measures between the competent authorities and individual landowners (EC 2000). Internationally, the Nature Directives acknowledge that threats to habitats and species are often of a transboundary nature, and both explicitly call for transboundary cooperative research between Member States.

Weaknesses

The Nature Directives do not set specific scales at which conservation action must be carried out.

3. EBM develops and uses multi-disciplinary knowledge

Strengths

The development of a protection regime for habitats and species, and designation of Natura 2000 sites, is done on scientific grounds and must consider element of biology, ecosystem functions and structure. A pre-defined list of habitats and species are set out in the directives. Assessment elements of their status, as defined in the HD, focus on natural habitat types (range, areas covered, specific structure and functions, future prospects) and species (range, population, habitat, future prospects).The effects of biodiversity loss, habitat fragmentation and ecological dynamics are considered.

The Nature Directives include consideration of social and economic issues, whereby Member States must provide information on threats and pressures for the assessment of conservation status for species and habitats (Art. 12 BD, Art. 17 HD). Measures taken under the HD and BD (Art. 2) must take into account economic, social and cultural requirements and regional and local characteristics of the area concerned which would assumedly entail multidisciplinary knowledge.

Weaknesses

There is no specific requirement to identify and consider key thresholds in ecological dynamics in order to maintain 'resilience'.

There is no explicit mention of the potential use of local knowledge in either directive.

4. EBM builds on social-ecological interactions, stakeholder participation and transparency

Strengths

Member States must take account of economic, social and cultural requirements and characteristics when implementing measures to restore the favourable status of sites and species.

Under the HD, any plan or project likely to have a significant effect on a Natura 2000, either individually or in combination with other plans or projects, shall undergo an appropriate assessment to determine its implications for the site. The competent authorities can only agree to the plan or project after having ascertained that it will not adversely affect the integrity of the site concerned (Art. 6.3). Projects can go ahead if there is no other satisfactory alternative, and there

are imperative reasons of overriding public interest, including those of a social or economic nature (Art. 6.1). In such cases the Member State must take appropriate compensatory measures to ensure that the overall coherence of the Natura 2000 Network is protected (Art. 6.4). Under the BD, Member States may also derogate in the interest of public health or safety, air safety, for the protection of flora and fauna and to prevent damage to crops, livestock, fisheries and water.

Thus, the Nature Directives allow for the consideration of various benefits that society receives from ecosystems. For example, the protection of a specific habitat or species is likely to help maximise associate cultural services (i.e., natural heritage) or the supporting services that a specific habitat or species provide for other ecosystem services. Derogations are allowed for maximising other types of services such as food provisioning services or provision of energy.

There is a general requirement for public participation and official EU guidance encourages Member States to involve the public, e.g. on issues related to the establishment of the conservation measures (EC 2012). At EU level, implementation is supported by the Habitats Committee (Art. 20 & 21 of HD) and the Ornis Committee (Art. 16 of BD) which comprise representatives from all member states and the EU Commission. Decisions are made with a qualified majority (using weighted votes).

Member States are also asked to reflect on positive changes in public acceptance towards biodiversity protection, and cooperation between authorities, nature conservationists and other interest groups and initiatives. Finally, there are several consultative bodies with stakeholders at EU level such as the Natura 2000 Biogeographical Process , which is a multi-stakeholder co-operation process managed by the EU Commission to enhance cross-territorial cooperation.

Weaknesses

The Nature Directives do not call for an explicit assessment of trade-offs in the provision of ecosystem services.

The Nature Directives do not require the active involvement of stakeholders. In particular, there are no requirements for public consultation and there is no indication of when it is appropriate to obtain the opinion of the general public. There is no legal requirement to include or confer with consultative bodies with stakeholders at EU level.

5. EBM supports policy coordination

Strengths

In terms of coordination of implementation between the BD and the HD, the protection regime for SCIs, SACs and SPAs has been harmonised through Art. 7 of the HD (Milieu et al. 2015). A change from a 3-year to 6-year reporting cycle for the BD means that the BD and HD are now reasonably synchronised so that information is available in policy-relevant cycles and can give strong input to the overall biodiversity debate. Both directives are characterised by a similar dual structure of measures, consisting of a network of protected areas and strict protection regime of species in the wider landscape. Similar steps are required (e.g., establishing conservation measures, preventing/mitigating impacts from plans and projects, managing them in accordance with ecological needs). Although management provisions of the HD (Art. 6.1) do not apply to SPAs, Art. 4.1 and 4.2 of the BD provide for a similar approach (EC 2000).

The HD requires adoption of prioritised action frameworks (Art. 8) to define the funding needs and priorities for Natura 2000 at a national or regional level and so facilitate their integration into different EU instruments, in particular financing ones.

Funding appears, thus, theoretically available and to a degree coordinated between different policy instruments. Only the LIFE programme provides dedicated support to biodiversity and Natura 2000 as a primary objective.

Weaknesses

Being anterior to the Water Framework Directive (WFD) and Marine Strategy Framework Directive (MSFD), there is no specific requirement in the Nature Directives to coordinate with the water and marine legislation.

Other EU funding instruments are primarily targeted to deliver EU goals on rural, regional, infrastructural, social and scientific development. The extent to which nature and biodiversity are successfully integrated into the funding programmes depends nevertheless primarily on priority-setting at national and regional levels and the capacity of stakeholders to absorb funds.

6. EBM incorporates adaptive management

Strengths

The Nature Directives establish processes that can support the implementation of adaptive management. For example, the Nature Directives require Member States to report progress on the state of conservation every six years. Member States have also a certain margin of manoeuvre or flexibility in implementing provisions. Under the HD, Member States can propose adaptations to the list of SACs in light of results of surveillance of conservation status of habitats and species (Art. 6). The concrete targets to be achieved can vary and can also evolve with for example better scientific knowledge. Finally, the HD stresses for example the need to go beyond simple management measures to ensure conservation towards preventive and anticipatory approaches to avoid deterioration, which can overall build ecological resilience.

Weaknesses

The Nature Directives do not require adaptive management but establish processes that can support its implementation. The requirement for Member States to report progress on the state of conservation every six years can encourage some cycles of planning and revisions, but it is not clearly spelled out in both directives.

Appendix ESM S2: EBM principles reflected in the Water Framework Directive

1. EBM considers ecological integrity, biodiversity, resilience and ecosystem services

Strengths

The key terms “ecological integrity”, “biodiversity”, “resilience” or “ecosystem services” are all implicitly reflected in the WFD. The key objective of the WFD is to achieve good status or potential for all water bodies by 2015 and avoid deterioration (Art. 4). Ecologic status is an expression of the quality of the structure and functioning of aquatic ecosystems associated with surface waters. It is defined as the deviation of specified biological elements from undisturbed reference conditions, supported by hydromorphological and physicochemical quality elements. Thus, the environmental objectives of the WFD consider aquatic biodiversity.

Through a classification of status into different classes (i.e., high, good, moderate, poor and bad status), the WFD considers the role of critical (e.g., pollution) thresholds and the need to maintain ecosystems within equilibrium and certain ranges to maintain resilience. Specific EU guidance is available on adaptation, although mainstreaming is not a requirement of the WFD.

It establishs an integrated framework for all European legislation pertaining to water, in particular bathing water, drinking water and wastewater treatment. Furthermore, recent emphasis in the Blueprint for Safeguarding Europe’s water has been on searching for multiple benefits, in particular with drought management through e.g. the concept of ecological flows (CIS 2015) and flood risk management through e.g. better environmental options for flood risk management (EC 2011) and the concept of Natural Water Retention Measures.

Weaknesses

None of the key terms “ecological integrity”, “biodiversity”, “resilience” or “ecosystem services” is mentioned in the WFD.

While the WFD aligns with the idea that all aquatic ecosystems should be protected, it does not aim to maximise all ecosystems services.

2. EBM is carried out at appropriate spatial scales

Strengths

The WFD recognises hydrological units, and sets the primary management units at the level of water bodies and the administrative unit at the level of river basin districts, first at national level and at international level if the river basin is transboundary. Furthermore, water bodies are discrete and significant parts of surface water, for example a river stretch or an estuary. The WFD recognises different water categories, including surface water bodies (i.e., rivers, lakes coastal, transitional) and groundwater bodies. Programmes of Measures (PoMs) can include measures targeting a specific water body to the whole river basin district. The WFD strongly promotes integrated water and land management, and therefore expands the traditional scale of water management from a sole focus on aquatic systems to surrounding land. In transboundary river basins, coordination among member states and with non-member states is explicitly promoted in the Directive.

Weaknesses

The scales promoted by the WFD –which are primarily the water body and river basin levels- may not always be appropriate to tackle the threats to the relevant aquatic ecosystem, for example when needing to tackle nitrogen deposition from air pollution (leading to water eutrophication) or when considering migratory fish with the open-seas.

3. EBM develops and uses multi-disciplinary knowledge

Strengths

The characterisation of the River Basin District (RBD) (Art. 5) includes an analysis of pressures and impacts from human activities, the economic analysis, the delineation of water bodies and the establishment of the typology and reference conditions for surface water bodies. The selection of measures has to take their cost-effectiveness ratio into account, and thus ensure compliance at minimum costs for both public and private entities.

Overall, the WFD requires the consideration of information from different economic sectors, and the assessment of status as well as the selection of measures mobilise knowledge from different scientific disciplines (e.g., ecology, chemistry, economy).

Weaknesses

The WFD does not explicitly require an impact assessment of the measures.

The WFD does not ask for a detailed understanding of ecosystem functions and structures, nor does it specify how stakeholder opinions and knowledge should be taken into account.

4. EBM builds on social-ecological interactions, stakeholder participation and transparency

Strengths

While the objective of good ecological status requires adequate attention to ecological needs, socio-economic concerns are considered in several ways. For example, good ecological status is not required, but good ecologic potential, for water bodies designated as "heavily modified" or "artificial" in view of their existing modifications to their hydro-morphology. The use of exemptions to reaching the environmental objectives (good ecological status and potential) is also possible if certain conditions are met. Exemptions include extension of deadlines (Art. 4.4), less stringent objectives (Art. 4.5), temporary deterioration (Art. 4.6) and new modifications (Art. 4.7).

Thus, the WFD allows for the consideration of environmental, economic and social factors which are associated with ecosystem services. For example, the maximisation of flood regulation services can be considered for justifying lower objectives. Strict conditions must be met for the use of designations and exemptions, and guidance by the Common Implementation Strategy (CIS) of the WFD has been published.

The WFD encourages the active involvement of all interested parties in implementation, in particular the production, review and updating of river basin management plans (Art. 14). Transparency is a strong requirement in the WFD as it specifically requires the open publication and distribution of timetables, work programmes, assessment reports, and draft plans. Six months consultation periods are requested. In addition, the public can access background document and information on request. Reporting to the European Commission is extensive, and includes assessment reports, monitoring programmes, plans and progress reports (Art. 15). Implementation at European level is supported by the CIS which consists of Member States and

stakeholder representatives. Multiple implementation guidance documents have been prepared and published by the CIS, including one specifically on public participation.

Weaknesses

The WFD does not foresee discussions about trade-offs between ecosystem services. The appropriate application of designations and exemptions has nevertheless been the subject of much debate. Substantial debate still exists on the practical interpretation of key methodological elements. This includes for example the characterisation of the hydro-morphological condition of water bodies, the application of the concept of overriding public interest or the use of disproportionate cost analysis.

While the WFD is explicitly supporting public consultation, decisions remain in the control of competent authorities. The degree to which consultation results are taken into account is largely left to Member States and competent authorities to decide.

5. EBM supports policy coordination

Strengths

The WFD specifically harmonises objectives and approaches across water-related policies by requiring the inclusion of relevant measures from other water directives in the WFD programme of measures. These take the form of basic or supplementary measures in the River Basin Management Plans (RBMPs).

The WFD generally requires that implementation should contribute to the protection of marine waters (Art. 1). The WFD provides more specific linkages with the Nature Directives (EC, 2011a). At the minimum, the WFD requires compliance with standards and objectives applicable under the nature directive (Art. 4.9). In particular, designation (i.e., as heavily modified or artificial) of a particular site does not change objectives under the Nature Directives. In addition, the application of exemptions under the WFD must be justified under the HD if the exemptions would significantly affect the conservation status of BD and HD protected species and habitats (Art. 4.8). In any cases, exemptions must be coherent with the measures taken under the Nature Directives (Art. 4.9). Recent initiatives at EU level such as Natural Water Retention promote integrated measures across the WFD and nature and other directives.

Fundamentally, Member States' PoMs should contain different instruments (legal, administrative, technical, infrastructure, training, etc.), and are potentially funded in different ways. Through the cost recovery provisions for water services (Art. 9, including environmental and resource costs), service users and polluters (according to the polluter-pays-principle) are expected to finance part of the measures. This will be complemented by public funds.

European funds – structural cohesion or Common Agricultural Policy (CAP) funds - can also contribute to finance some WFD measures. The Commission's proposal for 2014-2020 cohesion policy builds on key elements of the WFD proposing ex-ante conditionality for the use of cohesion and structural funds in the water sector. Cohesion policy provides an opportunity for joining water use management needs and implementation of water policy. In the current programming period of the LIFE programme, funding has been introduced with the possibility to co-finance projects which integrate different EU funds and other financial sources in a single, large scale project for the implementation of measures under the WFD. Within those, funding can be granted to RBMPs, Natura 2000 networks and cross-border flood protection strategies.

Weaknesses

Because the WFD is anterior to the MSFD, it does not create specific linkages with the MSFD.

In terms of financing, there are no specific funding sources linked to the objectives of the WFD.

6. EBM incorporates adaptive management

Strengths

Many of the WFD provisions support adaptive management. The WFD is organised around six year planning cycles, starting with the characterisation of the RBD, the monitoring and the assessment of status, the objective setting, and finally the PoMs and their implementation. Environmental objectives can be reach in up to three planning cycles (by 2027 at the latest) thereby allowing for a flexible, medium-term approach. Monitoring and evaluation of the effectiveness of measures link one planning cycle with the next.

The WFD provides for some flexibility with regards to the measures which can be included in the PoMs. Whereas the basic measures are fixed, a series of supplementary measures can be included, if deemed necessary for reaching the WFD objectives. Restorative and preventive measures are promoted, such as those for efficient water use and those preventing the impact of accidental pollution (Art. 11.3). These measures can increase robustness against risks and form part of a strategy to deal with uncertain future events.

The WFD mentions the precautionary principles and does not allow deterioration in the status of water bodies (unless exemptions apply) (Art. 1). Temporary deterioration in the status of water bodies is also allowed if it is the result of nature causes or exceptional circumstances which could not have been foreseen (Art. 4.6). Climate change can be integrated into the planning process (EC 2009).

Weaknesses

The WFD does not explicitly set out an adaptive management approach

The WFD does not integrate climate change in its legal text.

Appendix ESM S3: EBM principles reflected in the Marine Strategy Framework Directive

1. EBM considers ecological integrity, biodiversity, resilience and ecosystem services

Strengths

The MSFD explicitly refers within the legal text to the concepts of "ecological integrity", "biodiversity", "resilience" and "ecosystem services" (Art. 1.2, 1.3, 3.5, Annex I). The overall objective of the MSFD is to establish a framework to achieve or maintain Good Environmental Status (GES) in the marine environment by the year 2020 at the latest. GES is to be determined on the basis of 11 qualitative descriptors (set out in Annex 1 of the Directive), which should ensure that the marine environment is protected, preserved and, where practicable, restored. The ultimate aim is to maintain biodiversity and provide diverse and dynamic marine areas which are clean, healthy and productive. GES is associated with a situation whereby the structure, functions and processes of marine ecosystems allow those ecosystems to function fully and maintain resilience.

Of notable importance, Member States must apply the ecosystem-approach to keep levels of human activities compatible with the achievement of GES (Art. 1.3). A sustainable use of marine goods and services is sought. Marine Protected Areas (MPAs), which can contribute to ecological integrity, are expressly called for (Art 13.4). Measures included in Member States' PoMs must take GES descriptors into account, including biodiversity, ecological integrity, safe biological limits, etc. (Art. 5.b.i, Annex I). Types of measures proposed in Annex VI include input, output and spatial controls, which can be seen as measures to ensure activities are conducted within critical thresholds.

2. EBM is carried out at appropriate spatial scales

Strengths

The MSFD covers marine waters (the waters, seabed, and subsoil) of Member States' jurisdictional reach under UNCLOS and coastal areas (Art. 3.1). Environmental status includes factors that may affect the area both from within and outside the area concerned (Art. 3.4). The MSFD establishes marine regions that go beyond Member States' territorial boundaries. Member States should not only consider other nations' territories as extension of their own ecosystems, but should evaluate how they themselves affect marine areas that lie beyond their borders (Art. 13.8). There is thus much emphasis in the MSFD on transboundary cooperation from Member States (Art. 4; 5.1; 6), in particular regarding monitoring and implementation of measures (Art. 11.2; 7; Annex II).

3. EBM develops and uses multi-disciplinary knowledge

Strengths

The MSFD calls for Member States to undertake an Initial Assessment of the socio-economic features of their marine environments using existing data, which is considered a key part of the planning process (Art. 8.1). Planning steps include an analysis of pressures and impacts of the marine environment (Art. 8). Member States are further required to consider the social and economic impacts of measures to reach environmental objectives; Member States are required to carry out a Cost Benefit Analysis and should ensure that measures are cost-effective (Art. 13.3).

A Working Area on Cross-cutting Issues has been put in place in order to support project coordination, offer scientific advice and science-policy interface, and provide information on cost-effective measures as key area for all EU CIS Working Groups of the MSFD (EC 2013).

4. EBM builds on social-ecological interactions, stakeholder participation and transparency

Strengths

Member States are allowed to adopt derogations in the form of “exceptions” to reaching the environmental targets due to modifications or alterations to the physical characteristics of marine waters brought about by actions taken for reasons of overriding public interest which outweigh the negative impact on the environment (Art. 14.1). In addition, Member States are not required to take action if the costs to achieve GES are deemed ‘disproportionate’ to the determined risks (Art. 14.4). CIS guidance (EC 2015) has reviewed the topic of derogations and provides examples.

There are few reporting requirements to the European Commission under the MSFD. Member States are required to make information and data available to the European institutions (Art. 19.3) and to inform the Commission on the establishment of PoMs (Art. 13.9). In addition, Member States should make scientific information on MPAs as well as the intended affects of their PoMs in regards to the data available for the general public (Art. 13.6).

Member States must offer opportunities to interested parties to participate (Art. 19.1). A regulatory committee –the Marine Strategy Coordination Group (MSCG) - is established at European level although its role is not specified (Art. 25). The coordination group currently works as a platform to exchange information and encourage best practices, building on the WFD experience. The MSFD calls for multiple parties to be involved in its development and implementation, including bodies such as the Regional Sea Conventions, Advisory Bodies and Regional Advisory Councils that are already embedded in EU marine management, as well as land-locked countries that lie within respective catchment areas (Art. 6.1; 6.2; 19.1). Annex VI highlights that measures in PoMs could include measures for communication, stakeholder involvement and raising public awareness. Finally, the CIS suggests that the groups of people affected most by changes in ecosystem services from policies should be incorporated into the Initial Assessment (EC 2011a).

Weaknesses

Member States are not required to ensure a regular exchange with key stakeholders

5. EBM supports policy coordination

Strengths

The MSFD legal text explicitly makes reference to multiple policies and their coordination (Art. 13.2), such as the Urban Waste Water Treatment, the Bathing Water Quality Directives, as well as any forthcoming legislation on environmental quality standards in the field of water policy or international agreements. Types of measures suggested by the MSFD and supported by the CIS include management coordination measures (EC 2015a). In addition, the implementation of the Directive shall be supported by existing Community financial instruments in accordance with applicable rules and conditions (Art. 22). The most relevant funding sources are identified as the European Structural and Investment Funds, European Maritime and Fisheries Fund, European Rural Development Fund, LIFE and Horizon 2020.

Annex IV of the MSFD highlights that environmental targets must be compatible with existing commitments, including those under the Nature Directives and WFD. Thus, implementation of MSFD can contribute to achieving FCS, but cannot impair the implementation of the Nature Directives and the application of "exceptions" under the MSFD cannot take precedence over Nature Directives obligations (EC 2012a). In other words, FCS is a regulatory minimum under the MSFD (and can thus contribute to reaching MSFD environmental objectives). The MSFD requires the adoption of spatial protection measures which should include protected areas established under the HD and BD and international or regional agreements.

6. EBM incorporates adaptive management

Strengths

The MSFD explicitly incorporates adaptive management (Art. 3.5). Member States must regularly update their marine environment assessments, their targets for GES, monitoring programmes and PoMs every six years (Art. 17). This allows for adaptive management over time to respond to new or emerging marine threats and to adjust response measures accordingly.

The directive promotes a precautionary approach so that the capacity of marine ecosystems to respond to human-induced changes is not compromised (i.e., resilience) (Art. 1.3). Attainment or maintenance of good environmental status is seen as maintaining ecosystem resilience (Art. 3.5). The MSFD, thus, supports preventative and restorative measures, and the idea of no-deterioration and restoration is present throughout the directive. In this sense, the use of spatial protection measures in PoMs (Art. 13.4) can increase ecosystem robustness and adaptability.

Follow-up guidance suggests that sources of uncertainty should be explicitly identified, especially during the economic and social analysis (EC 2011a). Member States are allowed to identify instances where environmental targets cannot be achieved due to natural causes or force majeure, which allows for some flexibility to deal with unforeseen events.

Weaknesses

The MSFD does not set out an explicit approach to manage uncertainties, and Member States are not required to adopt mitigation measures to respond to expected long-term changes, such as climate change.

References

CIS, 2015. 'Ecological flows in the implementation of the Water Framework Directive', Luxembourg, ISBN 978-92-79-45758-6

EC (European Commission), 2000. Managing Natura 2000 sites. The provisions of Article 6 of the 'Habitats' Directive 92/43/EEC. Retrieved 26 January 2017 from: http://ec.europa.eu/environment/nature/natura2000/management/docs/art6/provision_of_art6_en.pdf

EC, 2009. CIS Guidance n 24 (2009). River basin management in a changing climate. Retrieved 26 January 2017 from: https://circabc.europa.eu/sd/a/a88369ef-df4d-43b1-8c8c-306ac7c2d6e1/Guidance%20document%20n%2024%20-%20River%20Basin%20Management%20in%20a%20Changing%20Climate_FINAL.pdf

EC, 2011. Towards Better Environmental Options for Flood risk management. A note from DG Environment.EC (European Commission) (2012). Frequently asked questions: links between the Marine Strategy Framework Directive (MSFD 2008/56/EC) and the Nature Directives (Birds Directive 2009/147/EEC (BD) and Habitats Directive 92/43/EEC (HD)): Retrieved 26 January 2017 from: http://ec.europa.eu/environment/nature/natura2000/marine/docs/FAQ%20final%202012-07-27.pdf

EC, 2011a. Communication from the Commission to the European Parliament, the Council, the Economic and Social Committee and the Committee of the Regions: Our life insurance, our natural capital: an EU biodiversity strategy to 2020. COM(2011) 244 final Retrieved 26 January 2017 from: http://eur-lex.europa.eu/legal-content/EN/TXT/PDF/?uri=CELEX:52011DC0244&from=EN

EC, 2012. "Commission Note on the Designation of Special Areas of Conservation (SACs)". Accessed online 2 November 2016: http://ec.europa.eu/environment/nature/natura2000/management/docs/commission_note/commission_note_EN.pdf

EC, 2012a. Links between the Marine Strategy Framework Directive (MSFD 2008/56/EC) and the Nature Directives (Birds Directive 2009/147/EEC (BD) and Habitats Directive 92/43/EEC (HD)). Retrieved 26 January 2017 from: http://ec.europa.eu/environment/nature/natura2000/marine/docs/FAQ%20final%202012-07-27.pdf

EC, 2013. Strategic document including a work programme for 2014 and beyond: "Learning the lessons and launching a re-enforced phase of implementation". DG Environment, Brussels. Retrieved 26 January 2017 from: https://circabc.europa.eu/sd/a/0514c9de-4a71-472b-adc6-d86ce3b23b95/ESA_13-2015_03%20MSFD%20CIS%20future%20work%20programme%202014-beyond.pdf

EC, 2015. Reporting on Programmes of Measures (Art. 13) and on exceptions (Art. 14) for the Marine Strategy Framework Directive. DG Environment, Brussels. Retrieved 26 January 2017 from: https://circabc.europa.eu/sd/a/aa788b20-badf-4125-87a7-08aba9633016/GD12%20-%20Guidance%20on%20Art%2013-14%20Reporting.pdf

EC, 2015a. "Mid-term Review of the EU Biodiversity Strategy to 2020". European Commission, Brussels. Retrieved 26 January 2017 from: http://eur-lex.europa.eu/legal-content/EN/TXT/PDF/?uri=CELEX:52015DC0478&from=EN

EEA (European Environment Agency), 2015. State of Europe's Seas, Report 2/2015, Copenhagen, European Environment Agency, 216 pp. Retrieved 26 January 2017 from: https://www.kowi.de/Portaldata/2/Resources/horizon2020/coop/EEA-State-of-Europes-Seas.pdf

Milieu Ltd, Institute for European Environmental Policy (IEEP), ICF International and Ecosystems Ltd, 2015. Evaluation Study to support the Fitness Check of the Birds and Habitats Directives. Draft – Emerging Findings. Retrieved 26 January 2017 from: http://ec.europa.eu/environment/nature/legislation/fitness_check/docs/consultation/Fitness%20Check%20final%20draft%20emerging%20findings%20report.pdf
